# Fertility preservation in Turkey: a global look for nationwide strategy development

**DOI:** 10.4274/jtgga.galenos.2019.2018.0116

**Published:** 2019-08-28

**Authors:** Şafak Hatırnaz, Kadir Bakay, Ebru Hatırnaz, Davut Güven, Alper Başbuğ, Önder Çelik, Gazi Yıldırım, Cihat Ünlü

**Affiliations:** 1 In Vitro Fertilization-IVM Center, Medicana Samsun International Hospital, Samsun, Turkey; 2 Department of Obstetrics and Gynecology, Ondokuz Mayıs University School of Medicine, Samsun, Turkey; 3 Department of Obstetrics and Gynecology, Düzce University School of Medicine, Düzce, Turkey; 4 Private Office, Uşak, Turkey; 5 Department of Obstetrics and Gynecology, Yeditepe University Faculty of Medicine, İstanbul, Turkey; 6 Department of Obstetrics and Gynecology, Acıbadem University Faculty of Medicine, İstanbul, Turkey

**Keywords:** Fertility preservation, oncofertility, oocyte-embryo freezing, treatment modalities, national program

## Abstract

As the reproductive technology advanced along with the improved outcome in cancer treatment demands implementing new fertility preservation, developing algorithms on fertility preservation requires tailoring for each society. Here, the authors attempt to modify the current medical literature on fertility preservation for the Turkish population. A PubMed search was conducted using the search term *fertility preservation*. Initially, 280 items of literature were accessed. In the second evaluation, 126 articles were examined and 154 items were discarded due to the low quality of the literature. In the final round, only 68 publications that were the most relevant were found eligible for inclusion in this review article. In order to develop a more systematic national guideline, forming a multidisciplinary approach to create a web-based network would be the first step. Both physicians and patients will have open access to the information. This database should be linked to an international consortium to stay integrated and open for updating. The aim of this review was to evaluate the relationship between the current situation in our country and the developments in the world in light of the literature, and to establish infrastructure for the development of future approaches in our country.

## Introduction

We, as doctors, must always keep in our minds the basic message from the Hippocratic Oath ***‘primum non nocere’***. However, every medical or surgical treatment carries certain degrees of side effects or complications, which may cause the deterioration of some functions while improving the others. Tremendous advances in medical diagnosis and therapy have increased the survival rates in children and young age women with malignancies. Thus, *fertility preservation* has become a must in the routine practice of oncology (1). This necessitates consultations with reproductive endocrinologists before and after the oncologic treatment (2). Developed countries started to arrange guidelines and established organizations and societies related to oncofertility and fertility preservation because the demand of consultation for fertility preservation became a serious matter. The Oncofertility Consortium (OC), supported by National Institutes of Health, was founded in 2007, and evolved as the largest organization for the improvement of fertility expectations of patients with cancer and medical professionals dealing with oncofertility worldwide (3). Nineteen countries are involved in the OC but only 6 organizations actively contribute to the OC, the others remain inactive. FertiPROTEKT, a strong organization for fertility preservation in Europe, expanded the indications and added severe rheumatic diseases and social indications, and also developed strategies and guidelines and recommendations for those diseases (4). Indications for fertility preservation apart from oncofertility include premature ovarian failure due to genetic reasons and autoimmune disorders such as diabetes mellitus, thyroid dysfunction, Addison syndrome, myasthenia gravis, Crohn’s disease, lupus, and rheumatoid arthritis (5).

Oncofertility can be defined as a new discipline hosting many medical and social disciplines, which aims to give cancer survivors an opportunity to preserve their potential to have baby. Fertility preservation comprises the efforts made to preserve the potential to obtain oocytes or embryos for future use by either surgical or medical methods in patients with cancer (6).

The current situation in Turkey is confusing. Turkey seems to be a member of the OC but it is not actively involved in OC activities. Oncofertility is an issue seems to have a place in gynecologic oncology and infertility congresses, but not more than that. There exists no official organization or society that deals specifically with fertility preservation. There appears to be no web-based program that to inform patients and medical professionals who deal with fertility preservation.

This review article aims to refresh the current knowledge on fertility preservation methods and to recommend what can be done in order to have a nationwide fertility preservation program in Turkey.

A PubMed search was conducted using the search term fertility preservation. About 280 items of literature were accessed and 126 of these literature items were subjected to a second evaluation. Sixty-eight publications were included in this review study.

## Discussion

Oncofertility is a multidisciplinary approach, and if it can be implemented in the same institution, it could be of great benefit. However, this is not a convenience that can always be present in all institutions at all times. Disorganization as well as detachment between the disciplines seems to be one of the fundamental problems related to the preservation of fertility. Another significant point is concerned with the evaluation of how much sensitivity physicians working in the field of oncology have. For this purpose, a pilot study conducted in the United States of America (USA) in 2009 revealed striking results. Sixty-one percent of the oncologists who participated in the survey stated that they always or most of the time explained the effects of oncologic treatments on fertility to their patients but 45% indicated that they did not refer their patients to an infertility specialist. The sensitivity of physicians who have previously attended a seminar on the subject matter of fertility preservation is higher than those who have never participated in such seminars (45% and 33%, respectively). Fifty-five percent of the physicians who participated in a seminar recommended the administration of a less aggressive chemotherapy, whereas this rate was determined as 29% for those who had not taken part in seminars. Patient attitudes, bad prognosis, and the immediacy required for the initiation of treatment seem to be the leading reasons why physicians are insensitive towards this issue. It is possible to consolidate the bridge between oncologists and infertility specialists further through increasing the number of training sessions as well as approaches that are geared towards enhancing sensitivity. Thus, fertility can be preserved in young patients with cancer whose survival rate has increased (7). A survey study conducted among hematologists in Turkey inquired about their attitudes and behaviors toward the preservation of fertility. Twenty-five physicians were contacted, and it was observed that all hematologists showed sensitivity towards fertility preservation; however, 8% of the participants stated that they were not aware of fertility preservation at all; 76% pointed out that they did not have sufficient knowledge of the subject matter; 88% of the physicians who responded to the survey stated that they wanted to be informed more about fertility preservation; and 23% suggested that a written brochure or written resource would be required on this subject matter. All the participating hematologists agreed upon the recommendation that Turkish Hematology Association should prepare a guideline on the subject and a sessions on fertility preservation should be held at congresses on a regular basis (8).

The differences in the physicians’ attitudes and behaviors pose an obstacle to the options for fertility preservation in cases where hematopoietic stem cell transplantation has been implemented. Accordingly, an invitation was sent to 1035 physicians in the USA, and only 185 of the physicians responded to the 29-question survey. It was revealed that the responding physicians had awareness as to the preservation of fertility, and having discussions over fertility preservation made them feel better. Yet, it was found out that only 55% of them referred their patients to an infertility specialist. Sixty-three percent of the participating physicians pointed out that their patients were so ill that they were not in the position of being able to postpone the transplantation. It was also maintained that the patients had natural barriers such as already being infertile during the onset of the treatment (92%). The study revealed that the demographic attributes of the physicians, and their knowledge and perception on the subject matter had predictive significance with regard to referring patients for the preservation of fertility (9).

Following a pilot study, Forman et al. (10) conducted a survey across the USA in 2010. They sent a questionnaire to oncology physicians three times in one year over the web- based SurveyMonkey system, requesting online responses from the participants of the survey. They received responses from 249 physicians out of 1701 questionnaires sent. Ninety-five percent of the physicians said that they discussed fertility preservation with their patients. Even though 82% of the physicians stated that they referred patients to an infertility specialist, only half of those patients attended such a consultation. Thirty percent of the physicians stated that they acted in an indifferent way regarding fertility while planning the treatment. It was observed that gynecologic oncologists attached much more importance to fertility compared with medical oncologists. In a similar vein, gynecologic oncologists considered preserving fertility by planning less aggressive treatments. The rates of oncologists who refer patients in academic hospitals are much fewer when compared with gynecologic oncologists. According to oncologists, patients can take the chance of having a 5% reduction in their survival rates for the preservation of their fertility (10).

New diagnoses and treatments emerge as a result of increasing genetic and epigenetic studies, as well as the revealing of the human genome (11). It is known that male infertility increases the risk of developing cancer in the future. The same applies for female infertility as well. Besides this, it is also thought that the medications used for female infertility may increase cancer risk. It is believed that infertility and cancer have common predispositions in terms of genetic and epigenetic aspects. Apart from these, common environmental factors also play a role in exacerbating these problems. Hanson et al. (12) studied that male infertility carries the risk of developing testicular cancer, bladder cancer, and thyroid cancer, as well as lymphoma and leukemia. The authors also observed that such a risk would also apply for their close relatives, concluding that a genetic common predisposing element triggered in germline cells could exist (12). Nagirnaja et al. (13) studied the genetic links between cancer and infertility, examining the known oncogenes and important genes in spermatogenesis. They inquired as to whether there was a link between these, having concluded that extensive genomic studies should be performed, and susceptible locations should be identified related to both infertility and cancer through germline scanning (13). James and Jenkins (14) determined that epigenetic changes in male infertility and cancer increase susceptibility for these two pictures. They also drew a conclusion through the two-hit hypothesis, that one epimutation causes infertility, while the other one leads to cancer.

A significant increase in the life expectancies of patients with cancer at young age has been observed owing to the novelties in treatments. The most frequently seen cancer types among young individuals aged 15-24 years in Europe are Hodgkin’s lymphoma, testicular cancer, and malignant melanoma (15). The 5-year survival among young patients is over 90%. The most commonly observed forms of cancer seen among adults aged 25-49 are breast cancer, colorectal carcinoma, cervical cancer, and malignant melanoma (16). The most frequently encountered malignancy among those aged below 35 years in the United Kingdom is breast cancer. Mortality rates in patients with breast cancer aged under 50 years have decreased significantly through the polychemotherapy approach. Nonetheless, aggressive chemotherapy and radiation therapy administrations are lamentably required for many frequently encountered cancer types, which may cause permanent damage of reproductive functions (17). This situation accompanies many others that have to do with quality of life, apart from the loss of fertility, including osteoporosis, depression, cognitive disorders, cardiovascular diseases, and sexual dysfunction. There is an increasing amount of interest in fertility preservation both among oncologists and also among reproductive endocrinologists and infertility specialists, which have brought about the production of many new treatment strategies. The preservation of fertility as a multidisciplinary approach was put on the agenda at the 2009 Evian Annual Reproduction Meeting (18).

No evidence exists as to the direct impact of cancer on the reproductive system, yet treatments thereof may bring about adverse effects in several locations. For instance, in cases where the entire body is exposed to radiation therapy during childhood with doses of 14-30 Gy, it is known that uterine growth and development slows down (19). Administration of uterine radiation therapy during childhood and the young youth period causes an increase in the frequency of miscarriage and intrauterine growth restriction in the future (20). The risks of acute ovarian insufficiency, premature ovarian insufficiency, premature menopause, low ovarian volume, and being of low weight in newborn babies were observed to be increased among patients with cancer who were exposed to radiation therapy and/or chemotherapy administered with alkylating agents (21). As for chemotherapy and radiation therapy, the target cells in the ovary are follicular, and this causes a huge amount of reduction in the follicles. In adddition, based on this situation, endocrine and reproductive functions deteriorate. The decreased primordial follicular pool raises the probability of ovarian insufficiency and premature menopause probability (17). The lethal dose for primordial follicles is 2 Gy (22). The gonadotoxic medication impact in the ovary causes a vicious cycle and follicle-stimulating hormone (FSH) release increases because the breakdown of primordial follicles reduces the secretion of estradiol and inhibin, which in turn leads to more follicles entering the cohort, causing much more follicular damage as a consequence (23). This point reveals that more sensitivity is required to be shown in the approach towards women with regard to the preservation of fertility. Premature ovarian insufficiency emerges at later ages and persistent amenorrhea is accepted as a marker of ovarian insufficiency (24). Checking the number of antral follicles (AFC) and antimullerian hormone (AMH) concentration before the initiation of the treatment and conducting follow-ups in the post-treatment period can be used as a marker for the detection of the harm of gonadotoxic treatment (25).

Radiation therapy and chemotherapy administered to the pelvic or spinal location is gonadotoxic and toxicity is concerned with either the mode of treatment or the relevant dose of the treatment (26). Chemotherapeutic agents are generally used in combination so as to benefit from their synergic effects and to achieve a more effective result on the tumor. The agents known to be the most gonadotoxic are those with an alkylating agent, which increase the cyclophosphamide toxicities in taxanes used in adjuvant treatments (27). Radiation therapy-induced damage is based on the dose, area of treatment, and frequency of its administration (20 Wallace 2005).

The highest gonadotoxicity is seen in cases when intensive combined chemotherapy and entire body radiation therapy are applied prior to bone marrow transplantation, in cases of metastatic Ewing sarcoma and soft tissue sarcoma, as well as in Hodgkin lymphoma in which alkylating agents are used (28).

Preservation of fertility should be recommended to young patients with cancer as early as possible; however, cancer treatment may take precedence over fertility preservation most of the time (29). It is recommended that patients should be consulted by an infertility specialist who should inform the patient accordingly so as to clarify the issue of fertility preservation (30). If there is the possibility and ample time for medical treatment, it could be tried out. If no such opportunity is present, then fertility-preserving cancer treatments should be considered. Fertility remains intact if medical treatment is administered in endometrial cancer or conservative modes such as radical trachelectomy are administered in the early phase of cervical cancer. Despite this, protection of the gonads from pelvic radiation and storage of the gametes and embryos should also be considered as alternative options (29,31).

The new oncology treatments provide the chance of leading a normal life to an increasing number of patients with cancer, particularly young patients. Such treatments also confer the opportunity of having children. Correspondingly, increasing achievements in assisted reproductive techniques (ART) have also boosted hopes, and the belief that cancer-induced and cancer treatment-induced infertility can be solved through medical approaches has been conceived. A significant proportion of young patients with cancer state that they cannot find the opportunity to discuss fertility sufficiently; some attribute this to cancer, whereas others attribute this situation to the scarcity of time (29,32). Most of the time, it is too late. Moreover, recommendations related to fertility preservation are often offered in an inappropriate manner and this overlaps with the period when the patients are overly confused with regard to their cancer treatments. This destabilizes the patients as a consequence. In some cases, a number of choices such as removing the ovarian tissue, breaking it up and implanting it under the skin have been developed; however, it has been observed that the right differentiation has not been made in terms of the presentation of these options. What is more, such works have been popularized dramatically by the media before the scientific findings have been revealed (33).

Freezing the ovarian tissue, urgent *in vitro* fertilization (IVF), *in vitro* maturation (IVM) and ovarian suppression by gonadotropin-releasing hormone (GnRH) analogues, and random start ovarian stimulations can be used as several methods for the preservation of fertility (34,35). An important issue worth taking into consideration at this point is the necessity of having an immediate discussion about two matters, which are cancer and the preservation of fecundity. It is for this reason that cancer and fertility-preservation matters need to be managed by adopting a multidisciplinary perception, putting forth all the possible choices and then determining the most appropriate approach. What is desired indeed is to form a “task force” in local medical committees that are competent in cancer and fecundity. For such local committees to be formed, it is necessary that organizations that are capable of administering all the aforementioned fertility-preserving approaches exist. There are insufficient numbers of centers on IVM and this situation seems to be a deficit. One of the important functions of task forces is that they closely follow studies on fertility preservation.

It is required to set the priorities and decide on whether to have a narrow or broad dimension for the formation of a committed “task force”. A task force with an inadequate dimension would fail to satisfy offering services, and a broad task force would experience difficulties in offering treatment options with a required level of sensitivity due to their increasing work burden. A sample study for such a task force was put into practice in Switzerland, in a French-speaking region of the country. An area with 1.5 million residents was chosen to be the pilot region (36). The number of patients with breast cancer (the most frequently encountered type of cancer that develops in one year and is seen among young women) was calculated. The results showed that 115 new patients among the age group below 45 years in such a population density emerged every year. Assuming that discussions about fertility preservation are made with the 50-70% of the young patients with cancer, it has been foreseen that the task force could only have contact with 60-85 of the patients. With the premise that patients with breast cancer account for 40% of young patients with cancer, it can be predicted that the total number of patients that the task force can see per year would be between 15 and 210 patients (when 1.5 million people are taken as the basis). Based on such data, it was concluded that such numbers could be at the threshold of low numbers for IVF centers, and the ideal target population density should be between 2-7 million in this regard (33).

At this point, this question may be addressed: do such patients become completely infertile or could they have a chance of spontaneous pregnancy?

The possibilities of natural conception through fertility-preservation approaches should be discussed with all patients with cancer. It is also important to act in line with the cancer type. As a general principle, it is known that primordial follicles are more resistant to chemotherapy compared with developing follicles. This situation also provides an explanation for the fact that patients menstruate 6-9 months after chemotherapy treatment. This period overlaps with the new development phase of primordial follicles from the primordial follicle pool.

Hematologic malignancies and particularly Hodgkin lymphoma come to mind when young age cancers are at stake; however, breast cancer appears to be the mostly encountered cancer during the reproductive period (at 13% during the reproductive period of a person) due to its prevalence (37). It is possible to observe spontaneous pregnancies following breast cancer treatment owing to the nature of the chemotherapies used in breast cancer. For this reason, it is of importance to bear in mind the high probabilities of conception in patients with breast cancer prior to identifying the fertility-preserving approaches. In addition to this, the fact that there will be a difference between menstruating and fertility periods should not be disregarded. Thus, checking AFC and AMH before cancer treatment and performing a reevaluation after the treatment can ensure the revealing of the dimension of ovarian reserve loss (38).

Despite having such possibilities, it is quite difficult to know who would be able to become pregnant and who would not be able to do so. However, ensuring fecundity is possible only through conception. Furthermore, chemotherapy agents that are used could have long-term effects and they may lead to infertility or menopause (39).

Ovarian functions and fecundity ameliorate following chemotherapy in breast cancer cases. It is generally seen in women in their 30s and there is a 3-6 week period between surgery and chemotherapy. For these reasons, the possibility of performing urgent IVF in the intermittent period emerges. Thus, it is important to avoid oophorectomy and the grafting of ovarian tissue as far as possible for patients in this age group, particularly in cases of breast cancer. Unilateral oophorectomy may cause FSH increase and premature menopause in patients in their 30s (40). Instead, administration of an urgent IVF and embryo freezing procedure could be opted for. In this period of 3-6 weeks, using aromatase inhibitors in ovarian stimulation also increases the probability of retrieving eggs and reduces exposure to estrogen (41). Another point to be paid attention to is that such an administration can be performed only on cases in which the patient first underwent surgery, and afterwards received chemotherapy. For patients with administration of neoadjuvant chemotherapy and subsequent surgery, such a treatment would not be preferred.

## Current strategies for fertility preservation in females

According to the data of the American Cancer Society, it is predicted that new cancer diagnoses were made for 790,000 women in 2012 (42). Eighty-three percent of the women aged below 45 years who were diagnosed as having cancer between 2002-2012 maintained their lives (43). The treatment of many types of cancer in the reproductive period of an individual involves either the removal of reproductive organs through surgery or the use of cytotoxic medications that partially or entirely affect the reproductive functions. Ovaries act as the target organs for cytotoxic treatments, and primordial follicles are affected directly by these treatments (44). The primary reasons why ovarian insufficiency develops after cancer treatments are dependent on the ovary reserve of the patient prior to the onset of the treatment, the dose of the treatment agent used, and its duration (45). Entire ovarian tissue freezing, ovarian cortical tissue freezing, ovarian transplantation, oocyte and embryo freezing, as well as using GnRH analogues happen to be several treatments planned. However, the treatment approach recommended by the American Society for Reproductive Medicine is the cryopreservation of the oocytes or embryos that are obtained by IVF (46,47). Other approaches are still regarded as experimental treatments. Controlled ovarian stimulation (COS/COH) is an approach of treatment that is preferred owing to its high success and efficacy rates (48). Many patients start their treatment without receiving any consultation about fertility preservation despite the time elapsed. Afterwards, cancer survivors have expectations about fertility. In the above parts of this review, the treatment choices for patients who are consulted and have contact with reproductive endocrinology and infertility specialists have been presented. Another point in question is how can patients who have expectations about fertility be treated after their targeted cancer treatments have proved to be successful? Cases of targeted cancer therapy enable the maintaining of cancer treatments while being able to sustain fertility-preserving approaches.

Freezing oocytes or embryos could be used for postpubertal patients and patients who are married. The possibility of performing this procedure is dependent on the following factors: the existence of an IVF center, having the competency of performing ovarian stimulation to patients with cancer, and being experienced in good embryo development and cryopreservation. This approach is no longer considered to be experimental (46). Data related to the egg freezing of patients with cancer and their pregnancies after treatment are highly limited. In recent years, randomized controlled studies in which pregnancies achieved through oocyte vitrification were compared with fresh oocyte embryo transfers reported that similar results were obtained in terms of implantation and pregnancy rates (48,49,50). For the time being, ovarian stimulation for the embryo or mature oocyte freezing is considered to be the most appropriate strategy for attaining pregnancy. This can be attempted if the following conditions are present: the patient does not have a situation that would prevent the collection of oocytes, there is available time for ovarian stimulation, the patient has a medical condition that is fit for this procedure and it is safe to perform ovarian stimulation. The most important problem at stake is that the patient is not on her menstrual period and the possibility that the treatment may cause delay. The AFC, AMH, and FSH levels have importance in determining the gonadotropin dose to be used (51). Short-term gonadotropin antagonist treatments could be preferred. However, in a situation where menstruation does not start, a mode of treatment independent of the menstrual cycle and that is even on luteal phase can be planned through random start protocols in order to avoid time loss (52,53). By taking into consideration the fact that the patients have the possibility of receiving treatment for themselves only, the most suitable treatment choice should be administered. On the other hand, it is important to avoid OHSS. The use of agonist triggers in antagonist cycles could be of benefit to serve this purpose (54).

Medical or surgical treatments can be performed conservatively, particularly for early phase tumors and borderline tumors in women; thus, fertility is preserved in this way.

For patients to whom local pelvic radiation therapy will be administered, as a result of ovarian transposition operation, the ovary can be detracted from the area where radiation therapy will have impact. In this way, it could be possible to preserve fertility. If it is planned to collect eggs following such an operation, transabdominal collection would be more apt.

All the treatments conducted for the purpose of fertility preservation other than those already specified are considered to be experimental treatments, this is particularly the case in the USA. Treatments that fall into experimental categories are stated below:

a. Ovarian tissue freezing

b. In vitro oocyte maturation (IVM)

c. Ovarian suppression by GnRH analogues

In some specific cases, there may exist an available time interval following the surgery of the patients, this time frame extends up until postoperative chemotherapy. For example, in patients with breast cancer who have undergone lumpectomy or mastectomy, there is a long period of time for chemotherapy following the surgery. The major concern here is the hypoestrogenic effect that will be induced by ovarian stimulation and also the emergence of adverse effects in the course of the disease due to ovarian stimulation. It is for this reason that gonadotropins can be used along with an aromatase inhibitor on such patients rather than being used alone (55). Similarly, the administration of bilateral prophylactic salpingo-oophorectomy (BSO) could be recommended for patients who are BRCA mutation carriers (56). Ideally, BSO should be performed after fertility comes to an end; however, there are alternative options for such patients such as the intermittent collection of oocytes and freezing the embryo or oocytes. In addition, PGD could be administered on these patients in the future, and through embryo transfer with the BRCA mutation discarded, it would be possible to prevent passing on the mutation to subsequent generations. Ovarian tissue transplantation is not recommended to BRCA mutation carriers.

Hematologic malignancies pose a serious problem to fertility preservation considering the thought that the course of the disease is severe and even a minor surgical intervention could cause a serious deterioration in the blood picture. Furthermore, even if the ovarian tissue is removed and can be transplanted subsequently, it is important not to overlook the probability that leukemia might be implanted once again through this tissue (57,58). Even though patients with lymphoma are more appropriate for fertility preservation, consultation is not recommended that much at the beginning because the treatments administered have minor gonadotoxic effects. For this reason, referral of patients in hematologic malignancies is done in cases of recurrence, or after chemotherapy or induction treatment, or prior to stem cell transplantation. Thus, patients have already started gonadotoxic treatment in hematologic malignancies (59).

The most sensitive patient groups in fertility preservation are children and adolescents. The determination of the appropriate strategy for these patients should be considered very carefully. It is harder to talk about this issue with patients and their families than one might anticipate. Besides this, fertility-preserving infrastructure does not exist or fails to be sufficient in children’s hospitals. It is possible to perform oocyte collection in postpubertal girls aged below 18 years. This option is also possible for peripubertal adolescents. IVM can also be recommended to such population.

## Other indications for fertility preservation

Fertility preservation is not only restricted to patients with cancer but can also be used in some other medical conditions (60). The indications of fertility preservation other than cancer are listed below:

a. Premature ovarian failure (POF),

b. Chromosomal and genetic abnormalities (Turner syndrome, 47, XXX, Fragile X GALT enzyme or FSH receptor mutation),

c. Autoimmune diseases (thyroid, polyglandular, multiple endocrine),

d. Environmental factors (malaria, varicella, Shigella may cause POF),

e. Surgical menopause (benign ovarian disease, prophylactic oophorectomy),

f. Cytotoxic agents for hematologic and autoimmune diseases,

g. Postponing fertility/social indications.

## Fertility preservation strategies in males

When compared with female cases, fertility preservation in males is slightly easier. Sperm freezing does not require any treatment beforehand, it does not cause time loss for the patient, and it is a simple procedure of giving a sample, which is also repeatable. Sperm cryopreservation is a male fertility-preservation method that is recommended on standard basis. It is important that semen samples have already been retrieved prior to chemotherapy and radiation therapy. At least 3 samples of semen are to be taken ideally and the storage should be performed by using many vials for the cryopreservation procedure. It could be hard to provide samples in young adults so it is important that they give the sample in an environment that is peaceful and comfortable. There are other challenges regarding the provision of sample, which are anxiety, fatigue, pain, additional morbidities, neurologic problems, diabetes mellitus, and hypogonadism. In such cases, the following approaches are recommended to be used to obtain samples:

a. Phosphodiesterase type 5, which is generally used in erectile dysfunction, but it is preferred in situations where giving sample is challenging (61),

b. Penile vibratory stimulation,

c. Electroejaculation,

d. Retrograde sperm collection and cryopreservation,

e. Cryopreservation of sperms obtained by surgery.

GnRH analogue treatment and the storage of testicular tissue from the prepubertal period for male fertility preservation are still considered to be experimental (62,63).

**Figure 1:**
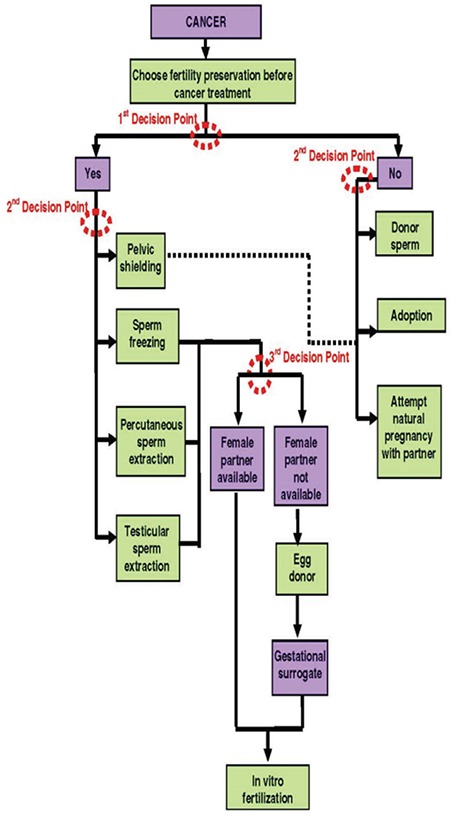
Decision tree for male oncofertility patient (with the permission of Theressa K. Woodruff)

**Figure 2:**
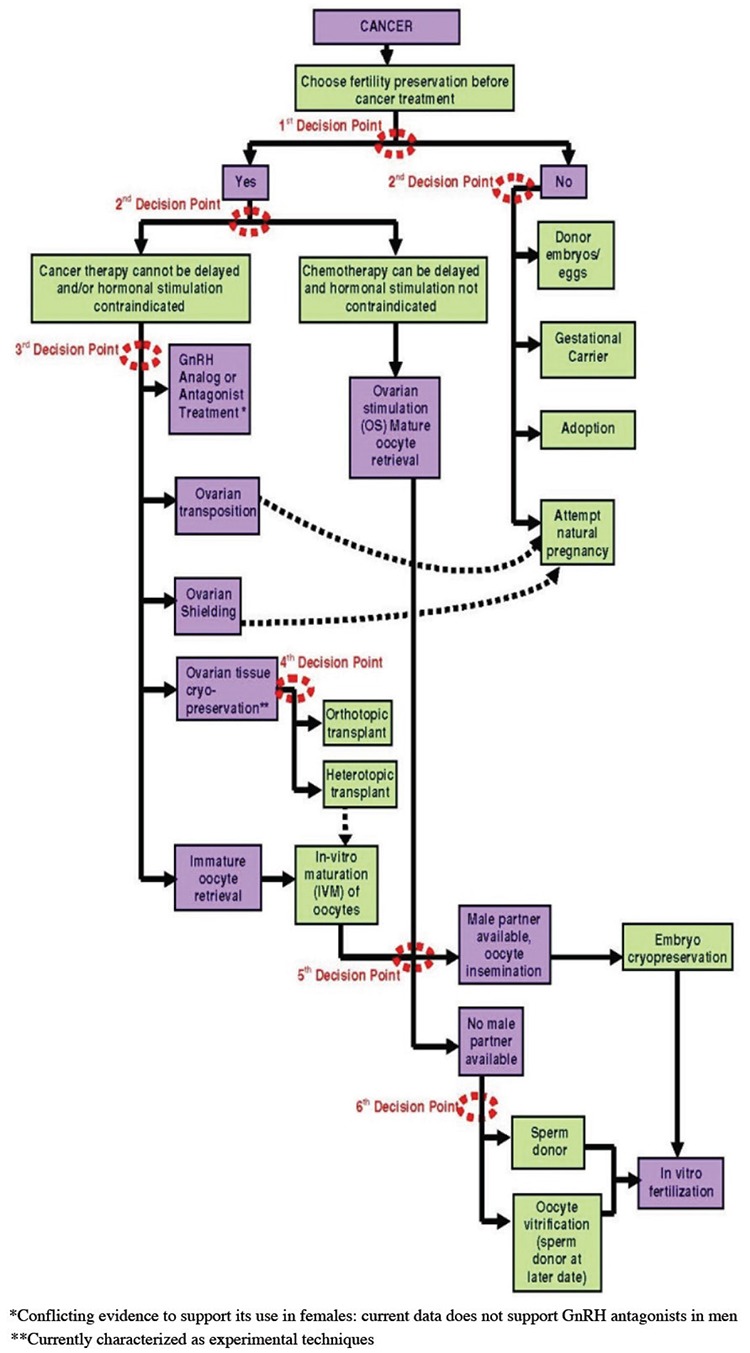
Decision tree for female oncofertility patient (with the permission of Theressa K. Woodruff)

When the effects of cancer on male fertility are analyzed, 30% of patients with testicular cancer demonstrate semen anomalies at the onset. Interestingly enough, semen problems at such a scale are also seen in patients who encounter other types of cancer at a young age. In a study conducted on 158 patients (aged 16-52 years) with Hodgkin lymphoma, it was revealed that 111 (70%) patients had degeneration in their semen parameters (64). Germinal epithelium is a highly sensitive tissue, and it is chemo-radiosensitive (65). Major subfertility is observed in cases where alkylating agents and radiation therapy are used. In radiation therapy administered with a dose that exceeds 4Gy, permanent fertility loss, namely sterility, is observed (20). Moreover, sperm tests, which show a downward trend within a period of 3-6 months after chemotherapy and radiation therapy, may start to get better slowly. When 2 years elapse following the treatment, spermatogenesis relapses in several phases with a probability of 97% and 94% after chemotherapy and radiation therapy, respectively (66). Azoospermia develops with a rate 59% in patients who are treated due to lymphoma, and its relapse duration is much longer (45 months) (67). Testicular somatic cells, namely Sertoli and Leydig cells, are more resistant than germ cells. However, alkylating agents or agents similar to those may affect sperm production by damaging these cells (Figure 1, 2) (68).

## Setting up a nationwide fertility preservation/oncofertility program in Turkey: Recommendations

The following recommendations have been put forth for preserving fertility and the efficacy of the oncofertility system concerning adolescents and young patients with cancer:

**a.** Dissemination of information, knowledge, training and available data,

**b.** Developing relations with the external centers, and being in contact with all the oncology units, family physicians, and nurses in places where multidisciplinary approach does not exist,

**c.** Establishing male-female fertility-preservation consultations and psychosocial support mechanisms through an internal referral system,

**d.** Generating referral forms, enabling the admission of patients from internal referral systems in other places,

**e.** Internal and external referral systems should keep in contact with one another periodically, hold meetings, and also perform professional updates,

**f.** Have robust database software,

**g.** Determining multimodal approaches that would offer maximum benefit, and physicians having discussions about these matters with their patients,

It is also very important to develop a record system related to fertility-preservation approaches administered to patients with cancer. It is recommended that such records be registered together with general records where ART data are collected across the country. The treatment approach administered on cancer type, rates of taking a baby home, and spontaneous pregnancy rates in similar cases are suggested to be noted in such records. Local fertility foundations should be involved in lobbying activities along with medical associations and Ministry of Health.

**h.** There should be liaison/contact points that serve the communication needs of the patients so that they can achieve results in a timely manner by accessing the points easily and also establishing prompt contact with the relevant physicians. Local task forces are also recommended to be established for this purpose.

**i.** Oncologists, reproductive endocrinologists, urologists, and surgeons competent in gonadectomy are required to act as part of an interdisciplinary medical team.

**j.** As the most important arm of this matter, centers of assisted reproductive techniques (IVF centers) that are competent and experienced in the area should exist. Such centers are expected to be qualified in fertility-preservation methods, stimulation protocols, oocyte freezing, embryo freezing, and IVM. Further competence is also required in regard to the freezing of both sperm and testicular tissue. There are directives in our country regarding sperm, egg, and embryo freezing. Ideally, these centers would be able to perform ovarian and testicular tissue freezing procedures even in prepubertal patients whose informed consent have been obtained. Such procedures are still accepted as experimental, however.

**k.** The support of mental health professionals should be taken in order to overcome the difficulty experienced by young adults, children or premenopausal patients when they are to make a decision. By performing genetic consultations, patients should be informed about passing the current disease on to the next generation genetically. One of the most crucial issues is making the financial situation clear and obtaining financial consultancy for this process, for which the government does not grant aid. In this way, approaches that will help to curtail costs could be identified.

**l.** Interdisciplinary collaboration is of crucial importance in fertility preservation. Patients should be referred to a competent reproductive endocrinologist or urologist after having a thorough discussion on the situation of the patient. If possible, all patients, including those at premenopausal age and adolescence, should undergo such a mechanism of referral. This is highly important so as to identify the optimal treatment and arrange the timing for fertility preservation. It is also important to eliminate legal and ethical problems along with professional arrangements.

**m.** When patients are referred to a reproductive endocrinologist, it is important to discuss at length all the medical and surgical options available for the preservation of fertility. It is also vital to talk about the existence of alternative treatment approaches such as donation and adoption, which are not legal in our country. The current situation of the patient should definitely be taken into consideration as to the decision. It may not be deemed appropriate to present matters related to fertility preservation to an individual who is too ill to be treated. The potential safety of future pregnancy after cancer treatment should be explained to the patient. Patients whose gametes and embryos are planned to be frozen should definitely be advised to go through scans for infectious diseases. Concerning patients who make the decision of freezing gametes, embryo, and tissue, what might lie ahead in the event of the death of the patient should also be discussed. This discussion should also be documented and recorded. If there is available time, patients are recommended to meet physicians, nurses, and mental counsellors.

## Further recommendations for a nationwide fertility preservation program

1. This organization must be controlled with a registration system by the Ministry of Health of Turkey.

2. It is recommended to be a part of the OC in a country-based program.

3. A web-based program should be implemented with the aid of the OC.

4. Societies related to oncofertility may be recommended to organize annual meetings to upgrade the knowledge concerning oncofertility and fertility preservation.

5. A nurse training program may be initiated by the Ministry of Health.

6. IVF centers experienced in IVM and ovarian tissue freezing need to be recognized and regionally selected centers and their staff must be trained for IVM and ovarian tissue freezing in order to establish regional centers for tissue and gamete freezing.

7. A multidisciplinary approach including oncologists, reproductive endocrinologists, embryologists, genetic specialists, radiologists and specialized nurses and social workers should be arranged for proper fertility preservation counselling.

8. Internationally accepted ovarian stimulation regimens should be implemented for IVF protocols.

9. Periodical multidisciplinary team counselling linked with task forces or satellite hospitals to manage the oncofertility patients in an appropriate manner.

10. Annual reports of the whole country together with registration of every single patient from the centers to the health ministry registration system.

11. Standardization of documents derived from the sources of OC should be carried out.

## Concluding remarks

Although there are centers dealing with oncofertility and fertility preservation individually, there is a strong necessity to have a nationwide registry that gathers all information from selected and accredited centers disseminated across all major regions in Turkey. For this, a colaborative study should be started with oncology societies, gynecology, and infertility societies, and of course the Society of Clinical Embryology, which may then be connected to the global Oncofertility Consortium to develop new strategies together with already experienced world centers that have been dealing with fertility preservation voluntariliy for many years.
